# Impact of temporal patterns in working contacts on epidemic spread

**DOI:** 10.1038/s41598-026-64555-z

**Published:** 2026-08-01

**Authors:** Aleksandr Bryzgalov, Johannes Ponge, Janik Suer, Tyll Krueger, Beryl Musundi, Chao Xu, Wolfgang Bock, Johannes Horn, Mahreen Kahkashan, André Karch, Julian Patzner, Mirjam Kretzschmar, Rafael Mikolajczyk, OptimAgent Consortium, Johannes Horn, Rafael Mikolajczyk, Carla Hartmann, Aleksandr Bryzgalov, Beryl Musundi, Chao Xu, Mahreen Kahkashan, Myka Sarajan, Julian Patzner, Hannah Derwanz, Johannes Ponge, Bernd Hellingrath, André Karch, Veronika K . Jaeger, Janik Suer, Huynh Thi Phuong, Mirjam Kretzschmar, Vitaly Belik, Andrzej K. Jarynowski, Marlli Zambrano, Steven Schulz, Richard Pastor, Alejandra Rincon, Ashish Thampi, Alexander Kuhlmann, André Calero Valdez, Lilian Kojan, Markus Scholz, Jan Pablo Burgard, Soheil Shams, João Vitor Pamplona, Wolfgang Bock, Lukas Bayer, Sudarshan Tiwari, Berit Lange, Isti Rodiah, Wolfgang Greiner, Maren Steinmann, Sebastian Gruhn, Uwe Siebert, Beate Jahn, Tyll Krueger

**Affiliations:** 1 Institute for Medical Epidemiology, Biometrics and Informatics (IMEBI), Interdisciplinary Center for Health Sciences, Medical School of the Martin-Luther University Halle-Wittenberg, 06097 Halle (Saale), Germany; 2 Department of Information Systems, University of Münster, 48149 Münster, Germany; 3 Institute of Epidemiology and Social Medicine, University of Münster, 48149 Münster, Germany; 4 Wroclaw University of Science and Technology, 50-370 Wrocław, Poland; 5 Faculty of Technology, Department of Mathematics, Linnaeus University, 351 95 Växjö, Sweden; 6 Department of Epidemiology, University Medical Center Utrecht, 3584 CX Utrecht, The Netherlands; 7 Interdisciplinary Center for Mathematical Modeling of Infectious Disease Dynamics (IMMIDD), University of Münster, 48149 Münster, Germany; 8 System Modeling Group, Institute of Veterinary Epidemiology and Biostatistics, Freie Universität Berlin, 14195 Berlin, Germany; 9 Machine Learning and Health Unit, Department of Engineering, NET CHECK GmbH, 10829 Berlin, Germany; 10 Institute for Social Medicine and Epidemiology, 23562 Lübeck, Germany; 11 Institute of Multimedia and Interactive Systems, 23562 Lübeck, Germany; 12 Institute for Medical Informatics, Statistics and Epidemiology, 04081 Leipzig, Germany; 13 Department of Economic and Social Statistics, Trier University, 54286 Trier, Germany; 14 Department of Mathematics, RPTU Kaiserslautern, 67663 Kaiserslautern, Germany; 15 Department of Epidemiology, Helmholtz Centre for Infection Research, 38124 Braunschweig, Germany; 16 Department of Health Economics and Health Care Management, School of Public Health, Bielefeld University, 33501 Bielefeld, Germany; 17 Institute of Public Health, Medical Decision Making and Health Technology Assessment Department of Public Health, Health ServicesResearch and Health Technology Assessment UMIT TIROL–University for Health Sciences and Technology, 6060 Hall. i.T., Austria

**Keywords:** Latent period, Spectral radius, GEMS, Redistribution of contacts, Week structure, Computational biology and bioinformatics, Diseases, Health care, Mathematics and computing

## Abstract

Most models for infectious disease spread simplify contact heterogeneity by assuming constant rates within a week. However, empirical studies show clear variation, such as reduced workplace contacts on weekends. In this work, we investigate the effects of daily variation in workplace contacts on the spread of respiratory infections using the individual-based framework GEMS (German Epidemic Micro-Simulation System) with a synthetic population of 5 million individuals. We compare the scenario with uniform daily contacts to the scenario with more contacts on workdays and fewer on weekends, keeping weekly totals constant. Simulations reveal that uniform contact rates yield higher prevalence for short latent periods (1–2 days) and lower prevalence for longer latencies (5–6 days). The effect diminishes for long infectious periods (*\ge* 7 days) but is more pronounced at lower basic reproduction numbers. Depending on the disease specification and the differences in weekday contacts, the impact of weekday heterogeneity can be very strong. These findings open new possibilities for weekday-dependent mitigation measures. We conclude that weekly contact dynamics should be explicitly incorporated into epidemic models to avoid systematic errors in reproduction number estimation.

## Introduction

**Fig. 1:**
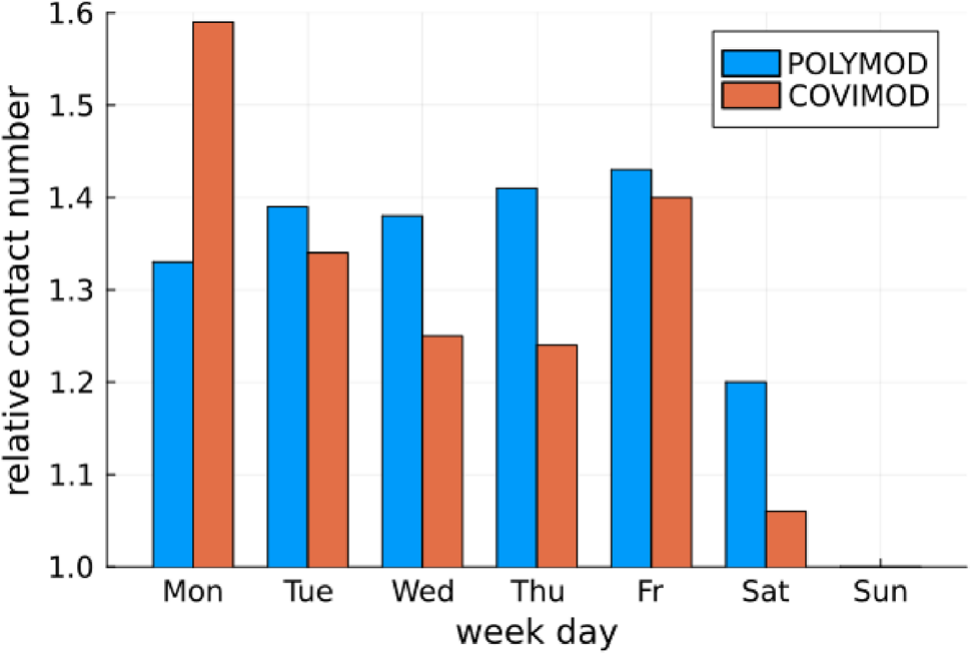
Relative number of contacts depending on weekday from POLYMOD study^6^ and COVIMOD Münster study^7^. In both datasets, the minimum number of relative contacts occurs on Sunday and equals 1.

Studies have shown that the dynamics of infection spread are specifically influenced by weekends^1–3^. This includes a clear reduction in workplace contacts and an increase in contacts due to leisure activities, such as social gatherings^4^ and increased mobility unrelated to work^5^. The POLYMOD study^6^ reported that the number of contacts on weekends was reduced by up to a factor of 1.43 compared with weekdays (see Fig. 1). Similar results were obtained in the COVIMOD study^7^.

Similar problems appear in areas such as information propagation in social networks^8^, resource distribution in supply chains^9^, social influence and opinion dynamics^10^, telecommunications and network congestion^11^, and collaborative workflows in organizations^12^. These problems are typically approached from the perspective of enhancing the spread, seeking optimized solutions to enhance the outcome.

**Fig. 2:**
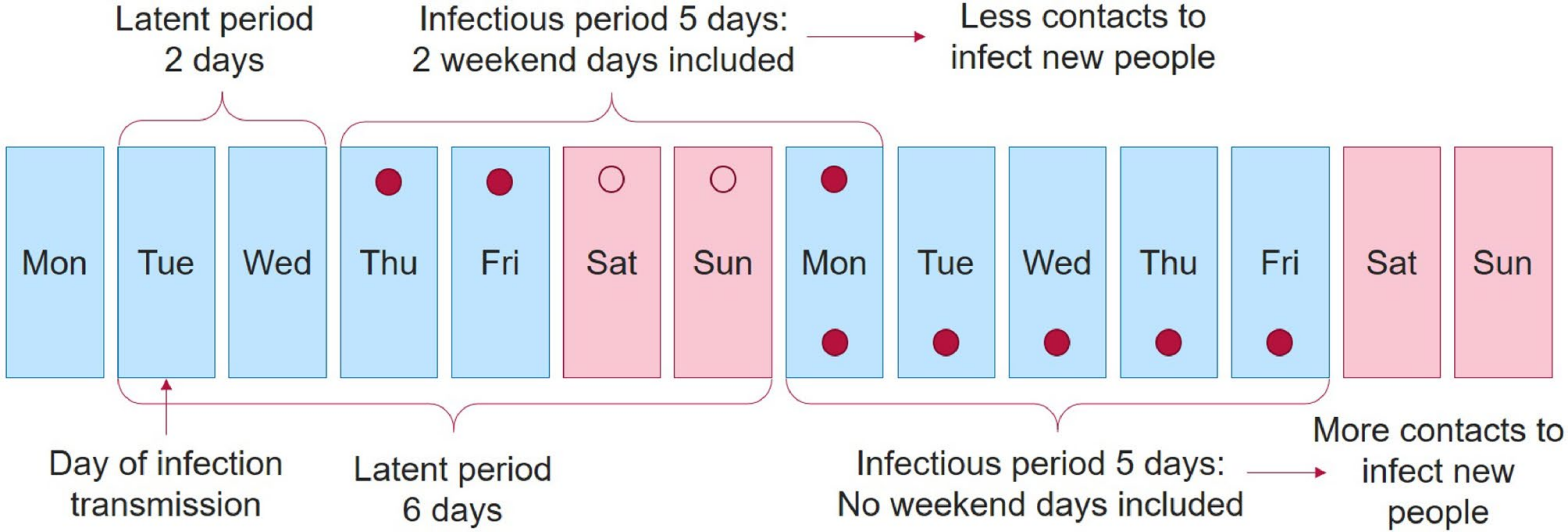
Example showing how the latent period affects infection counts depending on whether the infectious interval includes weekend days.

We focus on the consequences of the natural, uneven distribution of contacts throughout the week. How such heterogeneity interacts with the latent period and the duration of infectiousness is crucial for understanding outbreak dynamics. Consider the case where contact rates are very high on Friday but low during the weekend. If both the latent period and the infectious period are short, individuals infected on Friday will have limited opportunities to transmit further, as their infectiousness coincides with days of reduced contact. This example clearly illustrates that epidemic dynamics depend strongly on the weekly structure of contacts and the specific characteristics of the infectious period. In some cases, this can moderate or even halt the spread of infection due to a lack of transmission opportunities. Another example is shown in the Fig. 2, which illustrates how outbreak dynamics depend on whether the infectious period overlaps with the weekend. This effect is governed by the latent period *L*, as seen from the contrast between *L=2* and *L=6* days. The role of secondary infections in this mechanism remains less clear. However, transmission during weekends is generally lower due to reduced contact rates, and this reduction may interact with the latent period, leading to either amplification or suppression of the overall spread.

Classical compartment-based modeling using a weekly average contact rate produces similar predictions for the final epidemic size and the timing of the incidence peak, when compared to models that incorporate day-of-week contact patterns^1^. The use of agent-based models allows for the detailed analysis of daily variations in social contacts by explicitly representing structured populations and incorporating weekday- and location-specific contact patterns, thereby revealing possible differences between approaches based on averaged contacts and those that account for weekday variation.

The effect of variation in the latent and infectious periods has also been reported in^13^, where differences in epidemic peak timing and magnitude were observed using compartmental models. In that study, parameters corresponding to COVID-19 were used within an age-stratified framework (see Table 6 in^13^). The main conclusion is that the way the latent period is modeled, in particular its distribution, is crucial for accurately predicting the timing and magnitude of the epidemic peak. However, it does not provide a simple monotonic relationship describing how the peak changes with increasing or decreasing latent-period duration, but rather highlights a general sensitivity of epidemic dynamics to these assumptions.

A comparison between predictions using uniform contact rates and those using non-uniform rates could provide valuable insights into the following question: Can inaccuracies in the details of contact structure (such as averaging) lead to errors in modeling results? If so, does non-uniformity—or, conversely, uniformity—offer an advantage in controlling the spread of infection? Even when models provide a seemingly good fit to observed data, they may still yield misleading estimates of the reproduction number. In particular, a change in pathogen characteristics—such as a shift in the latent period between variants—can lead to substantial errors if the models rely on assumptions calibrated to earlier strains^14^. As a result, scenario modeling for emerging variants may produce incorrect projections, potentially causing wrong anticipation of outbreak dynamics and the effectiveness of interventions.

In^15^, the impact of contact-limiting strategies at workplaces, schools, and high schools was analyzed. More specifically, two approaches were considered: (1) rotating strategies, in which workers are evenly split into two shifts alternating on a daily or weekly basis; and (2) on–off strategies, where the entire group alternates between periods of normal in-person interaction and complete telecommuting. An open question is whether there are alternative ways to balance working time—and thus contact patterns—without reducing the total number of contacts, while still achieving a lower prevalence.

Indeed, certain non-uniform contact restrictions may offer advantages over uniform ones. Depending on epidemiological parameters, targeted restrictions applied on specific days of the week could prove more effective than uniformly distributed restrictions, while requiring fewer overall limitations. Concentrating restrictions on selected days may counterbalance synchronization effects between contact patterns and disease progression, thereby reducing transmission more efficiently. To our knowledge, this aspect has not yet been systematically addressed in the literature and warrants further investigation.

## Methods

As a modeling instrument, we have chosen an individual-based framework called GEMS (German Epidemic Micro-Simulation System)^16^. This framework incorporates the latest insights from the Poland MOCOS model^17^, Poland ICM Epidemiological Model^18^, and the Austrian full-scale agent-based model^19^.

### GEMS description

GEMS is an individual-based infectious disease modeling framework. It simulates the spread of respiratory pathogens within a synthetic population in discrete steps (e.g., days). GEMS operates using two core entities: individuals and settings. Each individual is assigned a set of attributes. Some attributes remain fixed throughout the simulation (e.g., ID number, age, gender, household ID, workplace ID), while others may change over time (e.g., susceptibility, disease state, symptom category).

Settings represent the environments where individuals interact. In this study, we used the following settings: household, workplace, school class (including kindergartens for children under six), and a global setting. The global setting aggregates all interactions outside households, workplaces, and schools, and allows any individual to come into contact with any other, unlike the other settings, where contacts are restricted to the “residents” of that specific environment.

Based on age, individuals are either assigned to a school/kindergarten or to a workplace. Pensioners neither study nor work and therefore do not have contacts in these specific settings.

Contacts are sampled independently for each individual on a daily basis. As a result, the set of contacts may vary from day to day, although repeated contacts with the same individuals can occur by chance. In structured settings with a limited number of possible contacts (e.g., small workplaces or households), repeated interactions are more likely. For example, in a workplace of four individuals, an individual with an average of three contacts per day will typically interact with the same colleagues across multiple days.

The number of daily contacts is drawn from a Poisson distribution with a specified mean (e.g., *3*), introducing stochastic variation in contact counts between days. Consequently, the number of realized contacts may differ from day to day. When the sampled number of contacts exceeds the number of available individuals in a given setting, contacts are assigned with replacement, allowing repeated interactions with the same individual within a single day.

Together, all individuals constitute the simulation population.

In the configuration used in this work, each infected individual progresses through the standard SEIR disease states: Susceptible (S), Exposed (E), Infectious (I), and Recovered (R). To simplify the model, reinfection (R *⟶* S transition) is not included.

At the start of each simulation run, a randomly selected subset of individuals is initialized in the exposed (E) state, while all remaining individuals are susceptible. A random day in the exposure interval is used. Susceptible individuals can become infected through contact with infectious individuals, with the transmission probability depending on the setting in which the contact occurs. New individuals are the earliest to be infected on the next day, due to the step-by-step nature of the process.

We focus exclusively on the contact setting (e.g., location) as the determining factor for transmission, assuming that transmission probability depends solely on the setting and does not vary with age or other individual characteristics.

### Population

**Fig. 3:**
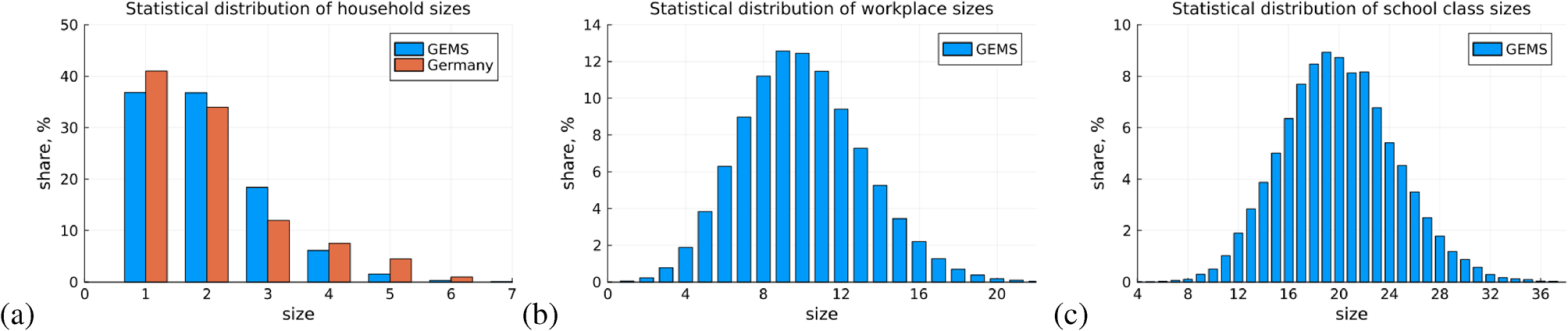
Distribution of households (**a**), workplaces (**b**), and school classes (**c**), generated by GEMS synthetically. In Fig. (**a**), the German distribution of households^20^ is shown additionally.

The population was synthetically generated based on predefined distributions of households, workplaces, and schools (including kindergartens). This flexible structure allowed us to isolate and assess the effects of weekday variations. In our study, we used a synthetic population of 5,000,000 individuals. The following sizes were specified for the settings:

- Households: we set the size following a zero-truncated Poisson distribution with a mean of 2, consistent with the average household size in Germany^20^ (see also Fig. 3a);
- Workplaces: we set the size following a zero-truncated Poisson distribution with a mean ranging from 2 to 100, depending on the specific task or simulation scenario;
- School classes and kindergarten groups: we set the size following a zero-truncated Poisson distribution with a mean of 20, consistent with the average school class size in Germany^21^;

An example of these distributions is provided in Fig. 3.

The age distribution was: 18.5% with 0 - 17 age (contacts in school and kindergarten), 66.5% with 18-64 age (working population), and 15% with 65 + age (elderly, not working population). The initial infected share was 0.1% of the population (5000 individuals).

### Disease progression parameters and infectivity per contact

Infected individuals progress through the SEIR disease states over time. To simplify the analysis, we define the latent period as the time between exposure and the onset of symptomatic infectiousness (Table 1).

For the basic pathogen model, we used an infectious period of *I = 5* days and a latent period of *L = 1* day, which are consistent with influenza A/H1N1^22^. On the Fig. 2, the example and relation between parameters are given. Additionally, the variant with a different latent period, *L = 6*, is shown. In our simulations, the day of infection transmission is considered to be fully included in the latent period.

In both the theoretical analysis and the selected simulation scenarios (the description will be further), we will present results not only for a basic pathogen model but also for varying latent and infectious periods. Among these parameter combinations, some correspond to values characteristic of SARS-CoV-2 infection (*L = 4*, *I = 10*)^13,23^.

**Table 1:** Description of disease progression from the^22^ and input parameters for the basic pathogen model that were used in simulations. In most simulations, we used exact values for *L* and *I* to have a pure impact.

Disease course stages	Description from^22^	Value used in GEMS
Latent period *L*	On average, viral shedding was detected 1 day after inoculation	1
Infectious period *I*	The mean duration of viral shedding was 4.80 days	5

As outcomes, we considered the prevalence during the simulation period, the peak value of infection cases, and the time of the peak. The simulation time was set to 500 days.

### Reproduction number

It’s important to underline that the reproduction number in the simulations is observable, not a parameter. The effective reproduction number $R_t$ is calculated based on the number of secondary cases $S_k$ that originated from any individual *k* belonging to set $E_t$, where $E_t$ denotes the set of infected individuals at time *t*. Dividing the latter by the former provides an estimate of the prospective reproduction number at day *t*:

1 $$\begin{aligned} R_t = \frac{1}{|E_t |} \sum _{k \in E_t} S_k . \end{aligned}$$

**Fig. 4:**
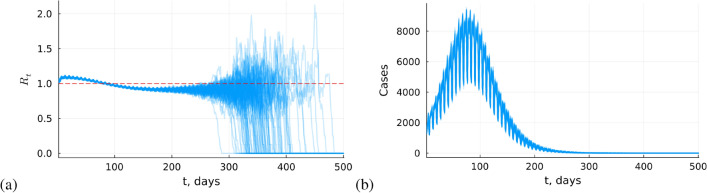
Example of (**a**) effective reproduction number $R_t$ curves from 100 simulations and (**b**) the corresponding daily new infection case curves. The high oscillations after 300 days are due to a mechanism for estimating $R_t$ (see formula (1)), which yields a better estimate as the number of infections increases. However, this requirement is not fulfilled after 300 days, and thus, the $R_t$ are not meaningful in that area.

Figure 4 illustrates a typical temporal evolution of $R_t$. The values observed during the initial time steps can be interpreted as an estimate of the basic reproduction number $R_0$, assuming complete susceptibility of the population and the absence of interventions–conditions that are satisfied in our simulations.

An alternative approach is to obtain an analytical estimate of $R_{0}$ from the next-generation matrix (described in the next subsection). In this framework, the spectral radius *ρ* provides an estimate of $R_{0}$. The corresponding *ρ* values are presented in the Results section and in the Appendices.

### Scenarios

As a “uniform” scenario, we considered a fixed number of contacts each day. In a “non-uniform” scenario, we redistributed the contacts, reducing them on weekends for children and the working population (only for workplaces, schools, and kindergartens). The reasoning behind this is clear: usually, people do not work during weekends, and children do not go to school or kindergarten. The total number of contacts was the same in both scenarios.

In the scenario with uniform contact rates, daily contact rates are assumed to be independent of the day of the week. The total numbers for a week are as follows: *3 × 7 = 21* contacts at the workplace, $6 \times {} 7 = 42$ contacts at school (including kindergarten), and *5 × 7 = 35* contacts in the global setting. See also Table 2.

**Table 2:** Mean number of daily contacts (daily contacts follow a Poisson distribution) and infectivity per contact. The global setting refers to all locations except those specified in the table columns. This combination of values gives an effective reproduction number of approximately 1.2.

Setting	Household	Workplace	School class/kindergarten	Global setting
Mean number of contacts per day *n*	Size - 1	3	6	5
Infectivity per contact *p*	0.0600	0.0300	0.0300	0.0132

In the scenario with non-uniform contact rates, we redistributed the contacts across weekdays while keeping the total number of contacts for each setting unchanged. See Table 3.

**Table 3:** Mean contact rates in the “non-uniform” scenario, following a Poisson distribution.

Setting	Workplace		School class/kindergarten		Global setting	
Week day	Workday	Weekend	Workday	Weekend	Workday	Weekend
Mean number of contacts per day *n*	4.2	0	8.4	0	5	5

The choice of the number of contacts per day *n* and the infectivity per contact *p* in each setting is determined by the following modeling assumptions. We distinguish the following transmission contexts: households, workplaces (including schools and kindergartens), and other locations (global setting). Contacts within households and workplaces tend to be relatively stable, as individuals repeatedly interact with the same groups, whereas contacts in the global setting are more heterogeneous. Consistent with previous studies^24^, we assume higher effective infectivity for contacts within households and workplaces compared to those in the global setting.

The number of contacts per day, *n*, is closely related to the infectivity per contact, *p*. In fact, the product $n\cdot p\cdot I$ determines the individual reproduction number, where *I* denotes the infectious period. Therefore, the values of *n* in this study should not be interpreted as directly observable contact rates. Instead, the combination of contact frequency and per-contact infectivity within each setting (household, workplace/school, or global setting), *n· p*, determines the relative contribution of each setting to transmission and thus the resulting distribution of secondary infections. In our case, the target distribution of infections was set to one-third within households, one-third within workplaces and schools (including kindergartens), and one-third in the global setting. This distribution represents a compromise between observed infection data^24–26^ and the relative number of contacts reported in empirical studies^6^.

One more important remark that we used sampling with replacement for contacts. This means that the probability of being infected in a smaller workplace is higher. Most simulations were done with a workplace size of 10.

### Estimation of expected number of infections by spectral radius

Previously, we have spoken only about the simulation opportunities, which themselves should be verified and validated. Such work was partially done, see, for example,^27^. To close this gap, we introduce an analytical method. It can be used as a preliminary evaluation to quickly assess the effects of temporal contact structures. Specifically, we can compare a uniform contact distribution with a non-uniform one—for example, cases with increased contact rates on working days—without running full simulations.

However, these evaluations rely on several assumptions: the method provides an estimate of the basic reproduction number for a single population group, it does not explicitly account for household contact structure, and the workplace size should be sufficiently large. The limitation in representing household contacts arises from the fact that only an average daily contact rate can be incorporated, while differences in household size are not explicitly resolved. In reality, contact numbers depend strongly on household composition (e.g., households of size 1 with no within-household contacts versus larger households with multiple potential contacts), which cannot be captured by a single averaged value. This limitation is not only related to the absence of contact distributions, but also to the inability to explicitly represent heterogeneity in household sizes. This also implies that GEMS simulations provide more precise results.

Since the working adult population constitutes the largest demographic group (66.5% of the total population), it is reasonable to choose this group for the analysis. We also conclude that the analytical estimates obtained for the working-adult population provide a qualitative indication of the trends expected in the simulation results for given daily contact rates, particularly in relation to changes in weekday contact patterns.

To calculate the expected number of secondary infections $N_{total}$ during the infectious period *I*, we use the spectral radius *ρ*. It is well known that the expected number of secondary cases produced by a typical infected individual during their entire infectious period in a completely susceptible population is mathematically defined as the dominant eigenvalue (i.e., the spectral radius) of a positive linear operator^28^.

We classify individuals into types according to the weekday on which they become infected. The matrix

$$M = (m_{ij})$$

specifies the expected number of type *j* individuals generated by a type *i* individual during its infectious period. Types do not differ intrinsically in their transmission characteristics; however, they influence the number of secondary cases through the temporal structure of contacts associated with the day of infection.

Thus, we can construct the matrix *M* based on the contact structures shown in subsection Scenarios (Table 2 and Table 3). Generally, each row of *M* represents the number of infectious contacts, depending on the day of transmission, the latent period *L*, and the infectious period *I*. For example, if transmission occurred on a Monday with a latent period *L=1* days and an infectious period *I=5* days, the corresponding row would look as follows:

$$\left( \begin{array}{ccccccc} 0&N_{tue}&N_{wed}&N_{thu}&N_{fri}&N_{sat}&0\end{array}\right) ,$$

where $N_{tue}$, $N_{wed}$,$N_{thu}$, $N_{fri}$, $N_{sat}$ are the corresponding number of infectious contacts on a certain weekday.

**Fig. 5:**
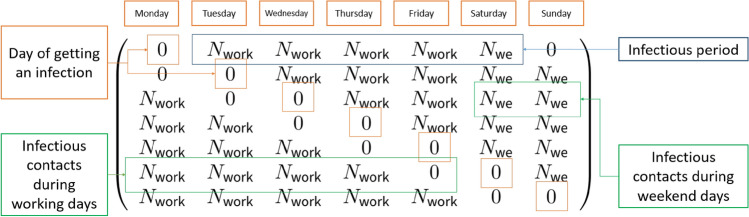
Next-generation matrix for the case *L=1* and *I=5*. The figure also indicates weekdays corresponding to the columns, as well as infectious contacts during working days and weekends; “0” denotes no infectious contacts. This may occur for two reasons: either the infectious period has not yet started, or it has already ended.

We also recommend looking at the Fig. 5 for a better understanding of the idea of creating a next-generation matrix.

To calculate the infectious contacts on a given day *N* we multiply the infectivity in a setting *p* by the corresponding number of contacts *n*:

2 $$\begin{aligned} N=n_{h}p_{h}+n_{w}p_{w}+n_{g}p_{g}. \end{aligned}$$

The index *h* is household, the index *w* is the workplace and the index *g* means global setting. Obviously, the values of *N* will not differ for the uniform distribution of contact rates and infectious period *I łe 7* days. For a longer infectious period, a certain weekday can be covered twice or more times, which results in a proportional increase of *N*. Also, we notice that *I=7;14;21;...* will not give any differences in *ρ* between scenarios, since the number of weekend days covered will be the same.

In the simplest case, we consider that the population is highly mixed and the contacts take place only in the global setting:

$$N=n_{g}p_{g}=n\cdot p$$

For a simplified explanation, we assign parameter values that reproduce the $R_{0}$ obtained in the detailed contact-based scenarios that include also household and workplace contacts. In a scenario with uniform contact rates, every individual has $n_{\text {uniform}}=3$ contacts per day. In the “non-uniform” scenario, on working days the contact rate is $n_{\text {work}}=4.2$ and on weekends $n_{\text {we}}=0$. Infectivity per contact is *p=0.075* to get the reproduction number greater than one. The corresponding numbers of infectious contacts will be: $N_{\text {uni}}=p\cdot n_{\text {uniform}}$; $N_{\text {work}}=p\cdot n_{\text {work}}$ and $N_{\text {we}}=p\cdot n_{\text {we}}$.

Let’s consider the infectious period *I=6* days (please, compare also with the case of *I = 5* on the Fig. 5) and the latent period *L=1* day. Thus, we can construct the matrices $M_{uni}$(uniform) and $M_{non}$ (non-uniform) of next-generation infections (see^28^) that can be written as:

3 $$\begin{aligned} M_{uni}=\left( \begin{array}{ccccccc} 0 & N_{\text {uni}} & N_{\text {uni}} & N_{\text {uni}} & N_{\text {uni}} & N_{\text {uni}} & N_{\text {uni}}\\ N_{\text {uni}} & 0 & N_{\text {uni}} & N_{\text {uni}} & N_{\text {uni}} & N_{\text {uni}} & N_{\text {uni}}\\ N_{\text {uni}} & N_{\text {uni}} & 0 & N_{\text {uni}} & N_{\text {uni}} & N_{\text {uni}} & N_{\text {uni}}\\ N_{\text {uni}} & N_{\text {uni}} & N_{\text {uni}} & 0 & N_{\text {uni}} & N_{\text {uni}} & N_{\text {uni}}\\ N_{\text {uni}} & N_{\text {uni}} & N_{\text {uni}} & N_{\text {uni}} & 0 & N_{\text {uni}} & N_{\text {uni}}\\ N_{\text {uni}} & N_{\text {uni}} & N_{\text {uni}} & N_{\text {uni}} & N_{\text {uni}} & 0 & N_{\text {uni}}\\ N_{\text {uni}} & N_{\text {uni}} & N_{\text {uni}} & N_{\text {uni}} & N_{\text {uni}} & N_{\text {uni}} & 0 \end{array}\right) , \end{aligned}$$

4 $$\begin{aligned} M_{non}=\left( \begin{array}{ccccccc} 0 & N_{\text {work}} & N_{\text {work}} & N_{\text {work}} & N_{\text {work}} & N_{\text {we}} & N_{\text {we}}\\ N_{\text {work}} & 0 & N_{\text {work}} & N_{\text {work}} & N_{\text {work}} & N_{\text {we}} & N_{\text {we}}\\ N_{\text {work}} & N_{\text {work}} & 0 & N_{\text {work}} & N_{\text {work}} & N_{\text {we}} & N_{\text {we}}\\ N_{\text {work}} & N_{\text {work}} & N_{\text {work}} & 0 & N_{\text {work}} & N_{\text {we}} & N_{\text {we}}\\ N_{\text {work}} & N_{\text {work}} & N_{\text {work}} & N_{\text {work}} & 0 & N_{\text {we}} & N_{\text {we}}\\ N_{\text {work}} & N_{\text {work}} & N_{\text {work}} & N_{\text {work}} & N_{\text {work}} & 0 & N_{\text {we}}\\ N_{\text {work}} & N_{\text {work}} & N_{\text {work}} & N_{\text {work}} & N_{\text {work}} & N_{\text {we}} & 0 \end{array}\right) . \end{aligned}$$

Columns reflect the weekday, and the rows display the day of infection transmission (zeros in matrices).

Then, the spectral radius is:

5 $$\begin{aligned} \rho =\max \{\lambda \in \mathbb {R}\mid \det (M-\lambda E)=0\}, \end{aligned}$$

where *E* is identity matrix and *λ* is eigenvalue.

Moreover, we derive the overall infected fraction in the population, i.e., the prevalence $a_{\text {total}}$. This quantity can also be interpreted as the probability (or likelihood) that a randomly selected individual becomes infected. By linking these two perspectives–population-level prevalence and individual infection probability–we obtain an analytical expression for $a_{\text {total}}$ further.

According to the general theory of branching processes^29^, the left eigenvector

$$\overrightarrow{V}=(v_1, v_2, \ldots , v_7)$$

of $(m_{ij})$, normalized such that $\sum v_i = 1$, gives the relative share of type *i* occurring in the asymptotics of the branching process. Furthermore,

$$M^{\star }\overrightarrow{V}=\rho \overrightarrow{V}$$

with *ρ* being a spectral radius. We claim that the relative proportions of the different types of individuals are still given by $\overrightarrow{V}$, since the types are not fixed a priori and no additional heterogeneity is assumed in the population. Even in the presence of a saturation effect, each individual has access to the same “pool of resources”, which does not alter the spectral radius or the corresponding eigenvector.

Let us consider a test individual drawn from a sufficiently large population with a given infected fraction. Each individual generates a Poisson-distributed in-degree. Thus, the likelihood that the test individual becomes infected, denoted by $a_{\text {total}}$, is given by:

$$1 - \exp \left( - \sum _i \sum _k v_k M_{i,k} a_{\text {total}} \right) .$$

Then

$$\sum _i \sum _k v_k M_{i,k} = \langle \overrightarrow{V}, M \overrightarrow{1} \rangle = \langle M^*\overrightarrow{V}, \overrightarrow{1} \rangle = \rho \sum _k v_k = \rho ,$$

where $\overrightarrow{1} = (1,1,..,1)$. Thus, we obtain for the prevalence $a_{\text {total}}$ the following formula

6 $$\begin{aligned} a_{\text {total}}=1-\exp (-\rho \cdot a_{\text {total}}) \end{aligned}$$

Using the values mentioned above $n_{\text {uniform}}$, $n_{\text {work}}$, $n_{\text {we}}$ and *p*, we calculate the spectral radii as $\rho _{\text {uniform}}=1.26$ and $\rho _{\text {non-uniform}}=1.35$ and corresponding $a_{\text {total}}$.

Additionally, we provide an estimation of the prevalence from the classical SEIR compartmental model^30^ using the $R_{0}=N_{\text {uni}}I$ and the results based on GEMS simulations. The comparison is shown in the Table 4.

**Table 4:** Comparison of estimations of prevalence (%) for a highly mixed population. The results are obtained by analytical formula (6), SEIR equation-based model and individual-based framework GEMS.

	Formula (6)	SEIR equation-based model^30^	GEMS
Uniform	46.9	47.1	46.9
Non-uniform	38.2	–	38.4

The generalization of matrices (3) and (4) for the different population groups (e.g., children, working adults, and the elderly non-working population) is not included in this work, nor is the extended form of formula (6). Further investigation is needed for a more precise assessment of household transmission in the calculation of the spectral radius.

Spectral radius estimations are included in the Results section and in the Appendices. We additionally demonstrate in Appendix 1 how the latent period *L* can affect the estimated spectral radius *ρ* and the associated disease dynamics, despite infection onset occurring on any day of the week.

## Results

In this section, we analyze the effect on the prevalence of the redistribution of contacts combined with variations of the latent period, infectious period, workplace size, and infectivity per contact. Also, we provide estimations based on the spectral radius (see the previous section). For each case within a given scenario, we conducted 100 simulations, calculating the mean values and standard deviations for the outcomes. The simulation time was set to 500 days.

We should also note that we did not observe substantial variability across simulation runs; the results were already stable with 100 realizations. Consequently, increasing the number of simulations did not meaningfully affect the reported outcomes, while only adding computational cost.

### Variation of latent period

In this section, we do not restrict ourselves to a specific type of pathogen; instead, we study the influence of the latent period on the results.

**Fig. 6:**
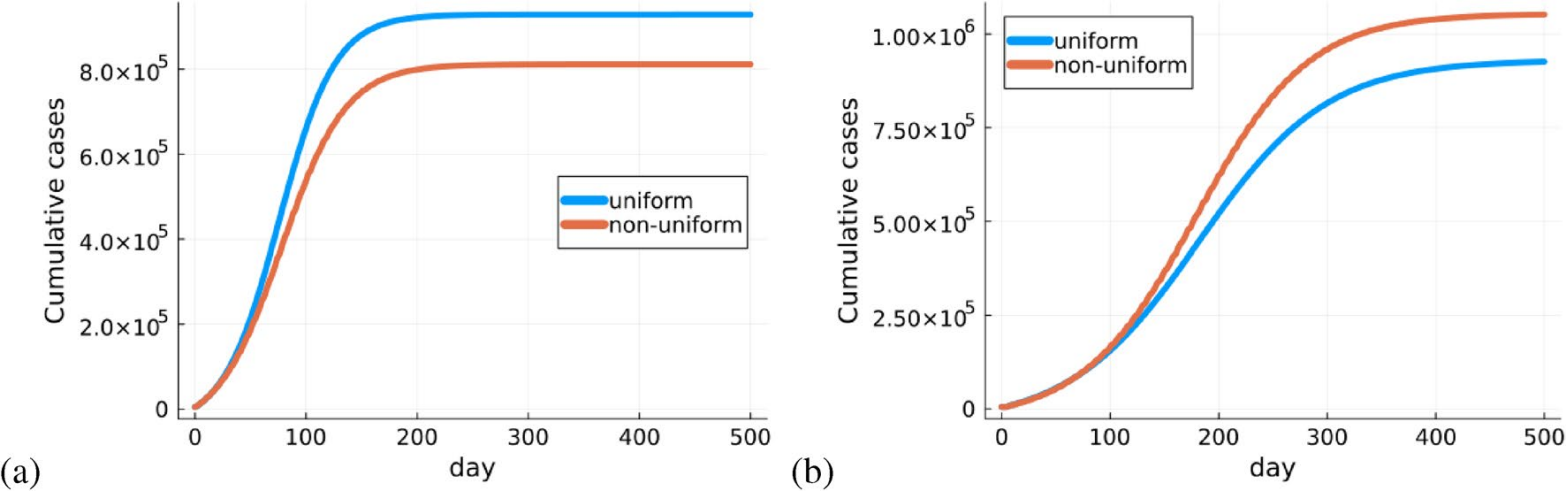
Cumulative cases for different scenarios. (**a**) latent period is 1 day and simulation time is 500 days; prevalences are: for uniform contact rates (18.58±0.15)%, for non-uniform contact rates (16.24±0.16)%. (**b**) latent period is 5 days and simulation time is 500 days; prevalences are: for uniform contact rates (18.52±0.15)%, for non-uniform contact rates (21.04±0.16)%. “±” denotes standard deviation. Intermediate standard deviation values do not exceed 0.3% and are excluded from the figure to improve visibility.

The latent period typically lasts several days before the infectious period begins. For a 1-day latent period, non-uniform contact rates lead to fewer infections than uniform contact rates (Fig. 6a). For a 5-day latent period, the pattern is reversed (Fig. 6b). This outcome was expected based on spectral radius calculations: the value of the spectral radius for the scenario with uniform contact rates and *L = 1* is $\rho _{\text {uniform}} = 1.08$, while in the scenario with non-uniform contact rates $\rho _{\text {non-uniform}} \approx 1.06$; for *L = 5* the corresponding spectral radii are $\rho _{\text {uniform}} = 1.08$ and $\rho _{\text {non-uniform}} \approx 1.10$.

For spectral radii of more combinations of *L* and *I*, see the table in Appendix 2.

In the simulations shown in Fig. 6, we used exact values for the latent and infectious periods. We set the workplace size following a Poisson distribution with a mean of 10.

To compare peak values and timing across the scenarios shown in Fig. 6, we use weekly aggregated case numbers. A comparison on a daily basis would be inconsistent, since the non-uniform contact scenario is, by definition, characterized by increased transmission during working days, which induces systematic weekday-specific fluctuations in incidence. In contrast, weekly totals provide an unbiased comparison, as the total number of contacts over the week is kept constant across scenarios. The corresponding comparison is provided in the table below.

**Table 5:** Peak values and peak times for the scenarios with uniform and non-uniform daily contacts.

Scenario	Uniform	Non-uniform
Peak time (*L=1*, *I=5*), week	11	12
Peak value (*L=1*, *I=5*), cases	67,246	54,048
Peak time (*L=5*, *I=5*), week	27	26
Peak value (*L=5*, *I=5*), cases	28,714	36,491

According to Table 5, an increase in the latent period shifts the peak to the right and reduces its magnitude. A similar tendency has been reported in^1^.

### Variation of workplace size

In the work^31^, a comparison is made between scenarios with proportional simultaneous scaling for all location types, including schools, workplaces, supermarkets, and social events. The considered range of workplace sizes is from 10 to 200. In contrast, we focused on the pure effect of workplace size variation, while keeping school class sizes and household sizes fixed.

Actually, the workplace size is the only parameter in our model for which precise empirical data are not available. In contrast, household structures and school class sizes are based on data representative of the German population^20,21^. We therefore explore a range of workplace sizes to assess how this parameter influences the outcomes. In all cases, workplace sizes are implemented as distributions (see Fig. 3), with the corresponding mean values reported in Table 6, which summarizes the GEMS simulation results for workplace size variation.

Under uniform contact rates, the number of contacts is fixed at 3, whereas under non-uniform contact rates, it increases to 4.2 on working days and drops to 0 on weekends. For schools, the corresponding values are 6 and 8.4, respectively, with no contacts on weekends. From the Table 6, it’s clear that there is a natural moderating effect for infection spread in small workplaces (i.e., those with fewer than 5 people). For very large workplaces (i.e., those with more than 40 people), the increase in infections reaches a saturation point. This outcome is expected, as the number of possible contacts in such large workplaces is high enough to allow the infection to spread more widely.

Additionally, in this subsection only, the values of *L* and *I* are assumed to follow a zero-truncated Poisson distribution rather than being fixed. Consequently, the values reported in Table 6 correspond to the respective mean values of these distributions.

**Table 6:** Prevalence (%), peak value (in cases per day), and peak time (week) for the scenarios with uniform and non-uniform contact rates for different workplace sizes. The sizes of other settings, including schools, remain unchanged. The transmission probability per contact and contact rates are taken from Table 2. Simulations were performed with latent period *L* having mean values of 1 and 5 days, following a zero-truncated Poisson distribution, and infectious period *I* having a mean of 5 days, also following a zero-truncated Poisson distribution. “±” denotes standard deviation.

Workplace size	2	5	10	20	40	100
Uniform contact scenario, *L* = 1						
Prevalence (%)	7.61 ± 0.20	13.58 ± 0.20	16.58 ± 0.16	18.35 ± 0.18	19.33 ± 0.15	19.95 ± 0.14
Peak value, cases per day	13,156	29,637	42,000	50,291	55,457	59,544
Peak time, week	13	15	15	15	15	14
Non-uniform contact scenario, *L* = 1						
Prevalence (%)	6.45 ± 0.23	12.22 ± 0.23	15.13 ± 0.19	16.85 ± 0.18	17.87 ± 0.18	18.34 ± 0.20
Peak value, cases per day	11,261	25,087	35,467	43,083	47,575	50,712
Peak time, week	10	15	15	15	15	15
Uniform contact scenario, *L* = 5						
Prevalence (%)	7.57 ± 0.20	13.57 ± 0.20	16.61 ± 0.20	18.37 ± 0.16	19.33 ± 0.15	19.94 ± 0.18
Peak value, cases per day	6,843	15,424	21,710	26,134	28,968	30,827
Peak time, week	24	29	29	28	28	28
Non-uniform contact scenario, *L* = 5						
Prevalence (%)	8.09 ± 0.20	14.28 ± 0.21	17.43 ± 0.20	19.31 ± 0.15	20.34 ± 0.16	21.00 ± 0.17
Peak value, cases per day	7,199	16,568	23,502	29,387	31,547	33,700
Peak time, week	28	31	30	29	29	28

The effect of the latent period is also evident: under uniform contact rates, higher prevalences (compared to non-uniform contact rates) are observed for all workplace sizes when *L = 1*, whereas lower prevalences are observed when *L = 5*.

We want to emphasize that the contacts were redistributed throughout the week only at workplaces and schools (kindergartens). Contacts in households and in the global setting remained fixed.

### Variation of infectivity

During the simulations, a stronger effect of contact redistribution is observed in scenarios with lower infectivity per contact *p* (see the last column in Table 7): the ratio of prevalence in the “uniform” scenario to that in the “non-uniform” scenario increases as *p* decreases. A similar effect is obtained by varying the infectious period *I* while keeping the infectivity per contact *p* and contact rate *n* constant, as shorter infectious periods correspond to lower reproduction numbers and reduced numbers of infections.

**Table 7:** Comparison of the workday/ weekend effect for the different infectivity on the prevalence. The results are given for workplace sizes following a Poisson distribution with a mean of 10. Contacts in the non-uniform contact scenario were changed according to the Table 3. Infectivity in settings (home, work, and global setting) was changed proportionally to save the corresponding infection distribution in settings. “±” denotes standard deviation.

In-household infectivity	Infectivity on workplace	Infectivity in global setting	Prevalence (%) uniform	Prevalence (%) non-uniform	Ratio (uniform/non-uniform)
0.0550	0.0275	0.0121	7.12 ± 0.25	5.19 ± 0.22	1.371
0.0600	0.0300	0.0132	18.58 ± 0.15	16.24 ± 0.16	1.144
0.0650	0.0325	0.0143	29.04 ± 0.12	26.96 ± 0.12	1.077
0.0700	0.0350	0.0154	37.95 ± 0.10	36.11 ± 0.09	1.051

### Interventions, based on contact redistribution

Redistribution of contacts can be used as an intervention strategy. We suggest considering the addition of a weekend day and a shortened week structure with two working days followed by two days off, while maintaining the same total number of weekly contacts. The effects are highly dependent on the latent and infectious periods; therefore, the choice of strategy should be more closely aligned with the characteristics of the pathogen.

One more important remark is that in the model, attendance at workplaces and schools is assumed to be synchronized: when adults go to work, children attend school or kindergarten. This assumption ensures consistency in daily contact patterns across population groups. If work schedules for adults are modified while school schedules remain unchanged, this synchronization is disrupted. For example, children may have days off while parents continue to work, leading to a mismatch in contact structures. To avoid such inconsistencies, school and workplace schedules are aligned in the considered scenarios.

In the case of an additional weekend day accompanied by a proportional increase in working time, we propose the following redistribution of contact rates (Table 8). The underlying idea is that working time should be increased proportionally to maintain an equivalent economic effect (while disregarding potential logistical restructuring within organizations). Our primary interest here is to compare scenarios with two versus three weekend days while keeping the total number of contacts unchanged.

**Table 8:** Contact rates in scenario with 3 weekend day working week. Corresponding weekly contact rates are: 21 contacts in the workplace and 42 contacts in the school.

Setting	Workplace		School class/kindergarten		Global setting	
Week day	Mon-Thu	Fri-Sun	Mon-Thu	Fri-Sun	Mon-Thu	Fri-Sun
Mean number of contacts per day	5.25	0	10.5	0	5	5

A week structure with two working days followed by two days off may be preferable for types of work that require round-the-clock coverage, rotating shifts, or continuous operations, including nights and weekends. The corresponding redistribution of contact rates is shown in Table 9. In this case, the “week” is shorter, consisting of four days in total. Consequently, infectious period durations divisible by four do not produce differences between scenarios (see the spectral radius estimations in Appendix 4).

**Table 9:** Contact rates in scenario with 2 working days / 2 days off working week structure. To obtain the numbers, we use the following: 21 contacts should be in a 7-day period in the workplace and 42 contacts in a school; the mean number of weekend days in a 7-day period is 3.5.

Setting	Workplace		School class/kindergarten		Global setting	
	Every 1-2 day	Every 3-4 day	Every 1-2 day	Every 3-4 day	Every 1-2 day	Every 3-4 day
Mean number of contacts per day	6	0	12	0	5	5

**Fig. 7:**
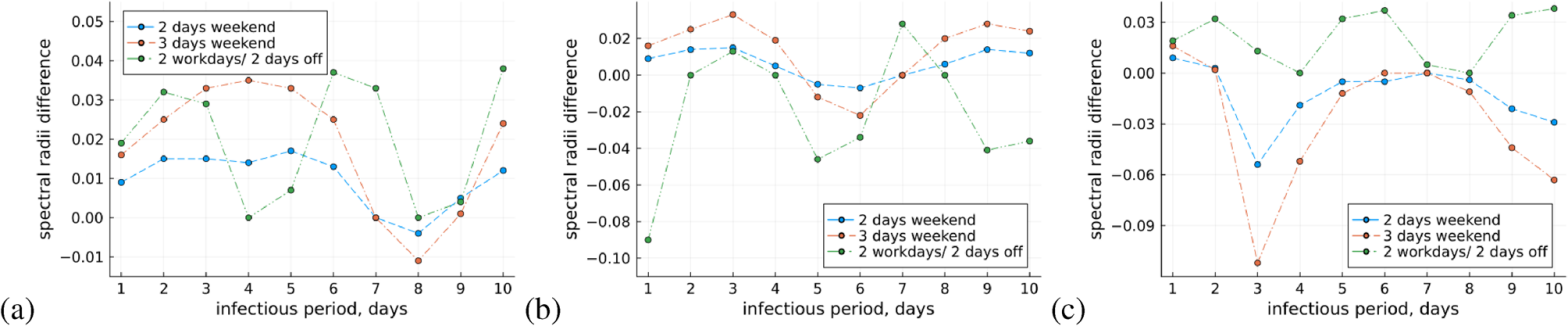
Spectral radii difference $\rho _{\text {uniform}}-\rho _{\text {non-uniform}}$. Non-uniform scenarios are: 2 days weekend - normal week with 2 weekend days, 3 days weekend - week with 3 weekend days, 2 workdays/2 days off - 2 working days, 2 days off. Weekly contacts are the same in all scenarios. (**a**) latent period is 1 day; (**b**) latent period is 4 days, and (**c**) latent period is 6 days. The used infectivity values are: $p_{h}=0.06$, $p_{w}=0.03$ and $p_{g}=0.0132$. This means for the values of *I<5* the corresponding $\rho _{\text {uniform}}<1$.

First, we provide a comparison of the numbers of infectious contacts in Fig. 7, based on spectral radius calculations for a single population group–working adults (see tables in Appendices 2–4). These estimations provide a qualitative understanding of how prevalence may change under different scenarios of contact redistribution. Based on this analysis, we then selected the parameters for the GEMS simulations.

For a latent period *L=1* day and infectious period *I<7* days (Fig. 7a), which generally reflects influenza-like dynamics, the additional weekend day can lead to a reduction in prevalence. For *L=4* days and *I=5* or 6 days (Fig. 7b), the results indicate that maintaining a standard week with two weekend days is preferable, as the introduction of an additional non-working day with reduced working contacts may increase prevalence. These parameter combinations may be interpreted as COVID-19-like dynamics with a shortened infectious period. This scenario may also reflect behavioral changes during infection, where symptomatic individuals reduce contacts and thereby effectively shorten their infectious period.

For a latent period *L=6* days (Fig. 7c), representing a hypothetical pathogen not directly corresponding to known respiratory diseases, redistributing the week into two or three weekend days generally leads to increased prevalence.

Guided by these findings, we selected specific combinations of infectious period *I* and latent period *L* for GEMS simulations. In particular, Case 1 (*L = 1* day, *I = 7* days) was chosen to examine whether changes in working-week structure can still produce an effect when, under the classical 7-day week, the uniform and non-uniform scenarios do not differ by default for *I=7*. Thus, a 4-day work week structure with 2 working days followed by 2 days off may have an advantage. Case 2 (*L=1* day, *I=5* days) was selected to represent an influenza case. Case 3 (*L=6* days, *I=3* days) was chosen to capture the largest differences in the results across different working-week structures. Infectivity per contact was adjusted to ensure that $R_0 > 1$. We summarize the GEMS simulation results in the Table 10.

**Table 10:** Comparison of the simulation results (prevalence, peak value, peak time) for 2 days weekend week, 3 days weekend week and 2 working days/ 2 days off week. Also, we provide data for the scenario with uniform contact rates. Case 1: *L=1* day, *I=7* days, $p_{h}=0.04$, $p_{w}=0.02$ and $p_{g}=0.0088$. The results for 2 days and for 3 days weekend are not different from the scenario with uniform contact rates, since the infectious period is 7 days. Case 2: *L=1* day, *I=5* days, $p_{h}=0.06$, $p_{w}=0.03$ and $p_{g}=0.0132$. Case 3: *L=6* day, *I=3* days, $p_{h}=0.09$, $p_{w}=0.045$ and $p_{g}=0.0198$. Exact values without distributions for *L* and *I* have been used. “ ± ” denotes standard deviation. All simulations were performed with workplace sizes following a Poisson distribution with a mean of 10.

Week structure	Case 1	Case 2	Case 3
2 days weekend	–	16.24 ± 0.16	14.46 ± 0.20
Peak value, cases per week	–	52,048	18,216
Peak time, week	–	12	32
3 days weekend	–	13.82 ± 0.20	24.30 ± 0.12
Peak value, cases per week	–	38,774	49,352
Peak time, week	-	12	25
2 w.d./2 days off	6.09 ± 0.21	17.66 ± 0.15	2.87 ± 0.16
Peak value, cases per week	10,288	61,785	5,000
Peak time, week	9	13	1
Uniform	9.16 ± 0.24	18.58 ± 0.15	5.08 ± 0.18
Peak value, cases per week	15,520	67,246	5,193
Peak time, week	15	11	3

The largest differences in spectral radii between scenarios are observed for *I=3* days, which corresponds to an enhanced transmission potential (see Case 3 in Table 10). In this regime, an alternating schedule of two working days followed by two days off may act as a potential mitigation strategy.

## Discussion

### Comparison of analytical and simulation frameworks

In this subsection, we formalize the relationship between the agent-based simulations and the analytical spectral radius approach, and outline the conditions under which the two frameworks are consistent or diverge.

We focus primarily on GEMS simulations as an agent-based model, complemented by an analytical approach based on the spectral radius. Ordinary differential equation (ODE) models are used only once, to demonstrate that in a highly mixed homogeneous population, all approaches yield very similar results.

The GEMS framework allows for substantially greater heterogeneity, including different population groups (age stratification), households, school classes, and workplace structures. This leads to more realistic dynamics, while the main qualitative insights are supported by spectral-radius-based estimations, here primarily applied to the “working adults” population group. Since working adults constitute the dominant group, the spectral radius approach is consistent with the GEMS simulations in terms of qualitative behavior (i.e., the sign of the effect). In particular, if the spectral radius indicates a higher total prevalence under uniform contact rates, the same tendency is observed in the GEMS simulations. However, the magnitude of the difference between scenarios may differ, as GEMS also includes children and elderly individuals, as well as additional structural factors such as household size distributions, which cannot be fully captured in the current theoretical framework.

The agreement between the two approaches may break down when the analyzed population group is not dominant. For example, if spectral-radius estimations are performed for elderly individuals, who represent a smaller fraction of the population, the GEMS simulations may yield a different qualitative outcome: the non-uniform scenario may result in fewer infections, while the spectral method predicts the opposite.

### Interaction of the infectious period and the structure of the working week

We conclude that a non-uniform distribution of contacts throughout the week can lead to either an increase or a decrease in epidemic outcomes, depending on the parameter regime. This pattern emerges from the interaction between the duration of the infectious period and the structure of the working week, specifically through the overlap between the infectious period and the weekly contact cycle.

For an infectious period of 7 days, both uniform and non-uniform contact scenarios effectively include two weekend days within the infectious window, resulting in no substantial difference in the total number of contacts experienced during the infectious period. For an infectious period of 6 days, differences between uniform and non-uniform contact patterns begin to emerge. In most cases, under the non-uniform scenario, two weekend days are included within the infectious period; however, configurations with only one weekend day are also possible, leading to variability in exposure. The strongest heterogeneity arises for infectious periods of 3–4 days. In this range, the number of weekend days falling within the infectious period can vary between 0, 1, or 2 with comparable probability, resulting in the largest variability in exposure to reduced-contact periods. For infectious periods of 1–2 days, the most likely outcome is that no weekend days fall within the infectious period, thereby further reducing the influence of the weekly contact structure.

Additionally, the effect of this temporal heterogeneity is strongly coupled with the latent period, which adds further complexity to the analysis. Consequently, these interactions should be taken into account in both agent-based models that simulate daily contacts and compartmental modeling approaches. At the same time, the magnitude of the effect depends on the epidemiological parameter regime and the underlying contact structure.

The strongest effects are observed when the effective reproduction number is close to 1 (see Table 7) and the infectious period is relatively short (typically *I < 7* days). In addition, pronounced differences between weekday and weekend contact rates amplify this effect; for example, a mean of 4.2 contacts on working days versus 0 on weekends in the workplace.

In this context, we considered several representative parameter regimes for the latent and infectious periods. The case *L = 1*, *I = 5* reflects influenza-like dynamics, whereas combinations such as *L = 4*, *I = 10* are broadly consistent with COVID-19–like infections (e.g.,^23^). More generally, the explored ranges *L = 1...7* and *I= 1...10* capture a wide spectrum of respiratory pathogens. The reproduction number in our simulations was not explicitly calibrated to a specific disease; instead, it emerges from contact rates and per-contact infectivity, corresponding to a regime with $R_0$ close to 1. This setting can be interpreted as a partially immune population, in which transmission operates near the epidemic threshold.

Thus, specific combinations of latent period, infectious period, and infectivity per contact can lead to qualitatively different dynamics under uniform and non-uniform contact structures, including regime shifts that resemble phase transitions (see Table 10). We do not exhaustively simulate all parameter combinations; however, approximate insights can be obtained using spectral radius calculations (see Appendices 2–4 and Fig. 7).

Finally, it should be emphasized that the observed effects depend on a set of coupled factors, which are summarized in Table 11.

**Table 11:** Summary of the effects produced by the given factors combined with contact redistribution.

Factor (parameter)	Range of values	Comment
Latent period	1-10 days	Defines how many weekend days will be included in the infectious period, which forms the key difference between the scenarios
Infectious period	1-10 days	The 3-4 day infectious period introduces more heterogeneity, resulting in differences between the scenarios with uniform and non-uniform contact rates; 7-day infectious period resulted in no differences
Workplace size	2-100 sizes	Saves the deviation between scenarios; for larger workplaces the relative difference is smaller
Difference between numbers of contacts in a given setting in workday and weekday	0; 3; 4.2; 5.25 contacts in workplace; 0; 6; 8.4; 10.5 contacts in school	When contact rates on working days and weekends are similar, the effect of redistribution is small; it becomes stronger as the difference between them increases
Infectivity per contact	0.055–0.070 in-household infectivity and proportionally changed in other settings (see Table 7)	Higher infectivity leads to increased prevalence, an earlier peak in daily cases, and a higher peak magnitude; simulations with smaller infectivity are more dependent on contact distribution

### Impact of workplace size on prevalence

The working adult population share is 66.5% and, 18.5% of children are in our simulations. But, of course, the changes that we applied for working contacts and school contacts in the “non-uniform” scenario do not affect the retirees (approximately 15%). Moreover, we must account for other contacts (household or global setting), which remain the same and can support the spread after the spike.

We did not perform simulations with a fixed workplace size, instead, we simulated distributions of workplace sizes (Fig. 3). However, we do not expect qualitatively different effects in that cases, as heterogeneity in workplace size is likely to average out: larger workplaces tend to be offset by smaller ones, leading to transmission dynamics that are effectively governed by the mean workplace size.

We also conclude that the effect of workplace size is much stronger than the redistribution of contacts during the week (Table 6).

### Adaptation of working week structure to the type of the pathogen/ respiratory infection disease

Additionally, we have considered scenarios that can support a specific kind of intervention, balancing the working time. The general idea is to leverage the advantages of contact redistribution, depending on the pathogen or disease type.

The provided method for estimating the spectral radius can be used as a preliminary qualitative result.

Of course, an implementation of studied strategies raises several practical challenges related to working norms (e.g., maintaining total working hours while reducing the number of working days), organizational constraints, and logistical issues. At the same time, such a structure may be feasible in certain sectors where longer shifts are already possible, such as industrial production or hospital settings. In addition, the effect could potentially be enhanced by splitting working groups across different working schedules, thereby further reducing repeated contacts within the same subgroups.

Such a redistribution of working time may also not be equally acceptable for all employees, as preferences regarding work–life balance differ. While some individuals may prefer longer working days combined with more days off, others may favor shorter working days distributed over a larger number of working days per week.

### Possible generalizations

We identify several directions for future work:

- We employed a completely synthetic population, which allowed us to treat workplace size as a tunable parameter. A promising avenue for future analysis is the comparison of detailed, fitted simulations of the temporal dynamics of the reproduction number between models that explicitly incorporate dynamic contact rates and those that instead rely on averaged contact patterns.
- Real-world contact distributions across the week are more complex than the simplified scenarios considered here^6,7,32^. Future work could incorporate empirically derived weekday–weekend contact structures and alternative forms of contact redistribution, which may lead to stronger or qualitatively different effects.
- We did not consider a U-shaped distribution of workplace sizes. Nevertheless, we expect similar qualitative effects, given the observed correlation between workplace size and infection outcomes.
- Reinfections (i.e., transitions from recovered to susceptible) were not included, as they were not expected to have a major impact. In most simulations, prevalence after 300 days closely matched the values observed at 500 days. Naturally, reinfection dynamics should be incorporated in future work to investigate the endemic equilibrium state.
- The model could be extended to account for heterogeneous vaccination coverage across different age groups^33^. In particular, age-structured immunity may interact with temporal contact patterns and modify the impact of weekday–weekend heterogeneity on transmission dynamics.
- Variation in household sizes and school class sizes could also be explored.
- In the current formulation, the global setting aggregates all contacts that are not explicitly assigned to households, workplaces, or schools. While this simplification enables tractable modeling, it inevitably masks important heterogeneity in social interactions. A more realistic representation would distinguish between different types of social settings, such as leisure activities and public events, and account for their specific characteristics, including age composition, contact intensity, frequency, and environmental context (e.g., indoor versus outdoor). For example, sporting events, concerts, and religious gatherings differ substantially in both participant structure and contact dynamics. In addition, variation in social activity across population groups–for instance, the typically higher contact rates among college students compared to older individuals–may further influence disease dynamics. Incorporating such distinctions represents an important direction for improving the realism and interpretability of the model.
- The influence of the distributional shape of the latent and infectious periods merits further study. In this work, we primarily considered fixed values and employed a zero-truncated Poisson distribution only in simulations with workplace size variation (see the corresponding subsection in the Results). Alternative distributions may affect the results in different ways.
- We did not consider asymptomatic disease progression, despite its relevance for epidemic control^34^. Future work should investigate how asymptomatic transmission interacts with age-dependent susceptibility^35^, self-isolation behaviour, and contact redistribution, in order to obtain more accurate estimates of epidemic dynamics.
- Regional heterogeneity represents another important factor. For instance, analyzing infection spread in bordering regions with differing working-week structures would provide an interesting case study.

## Supplementary Information

Supplementary Information.

## Data Availability

The datasets generated during and/or analysed during the current study are available from the corresponding author on reasonable request. The simulation code and model configuration files used to produce the results are also available from the corresponding author on reasonable request.
