# Supplementary material for: Impact of temporal patterns in working contacts on epidemic spread
Source: Sci Rep. 2026 Aug 1;16:23725. doi: 10.1038/s41598-026-64555-z (PMC13428738; doi:10.1038/s41598-026-64555-z)
Supplement: Supplementary file 1 — Supplementary Information. [file 41598_2026_64555_MOESM1_ESM.pdf]

## Appendix 1. Spectral radius calculation example for the case with contacts in three settings

In a case with contacts in three settings (household, workplace, and global setting), we can provide an upper bound for $R_0$, even when assessing a single dominant population group. We performed this estimation for working adults, assuming that each working individual has one household contact. This results in the number of infectious contacts on a working day being: $N_{\text{work}} = n_h p_h + n_w p_w + n_g p_g = 1 \cdot 0.06 + 4.2 \cdot 0.03 + 5 \cdot 0.0132 = 0.252$ and on weekend day $N_{\text{we}} = 0.126$. The values are taken from Tab. 2.

Moreover, it can be shown why the latent period $L$ determines how many weekend days fall within the infectious period, since infection onset can occur on any day of the week. Comparing the matrices $M_{non,L=1}$ and $M_{non,L=5}$ for $L = 1$ and $L = 5$, respectively (with $I = 5$):

$$M_{non,L=1} = \begin{pmatrix} 0 & 0.252 & 0.252 & 0.252 & 0.252 & 0.126 & 0 \\ 0 & 0 & 0.252 & 0.252 & 0.252 & 0.126 & 0.126 \\ 0.252 & 0 & 0 & 0.252 & 0.252 & 0.126 & 0.126 \\ 0.252 & 0.252 & 0 & 0 & 0.252 & 0.126 & 0.126 \\ 0.252 & 0.252 & 0.252 & 0 & 0 & 0.126 & 0.126 \\ 0.252 & 0.252 & 0.252 & 0.252 & 0 & 0 & 0.126 \\ 0.252 & 0.252 & 0.252 & 0.252 & 0.252 & 0 & 0 \end{pmatrix}$$

and

$$M_{non,L=5} = \begin{pmatrix} 0.252 & 0.252 & 0.252 & 0 & 0 & 0.126 & 0.126 \\ 0.252 & 0.252 & 0.252 & 0.252 & 0 & 0 & 0.126 \\ 0.252 & 0.252 & 0.252 & 0.252 & 0.252 & 0 & 0 \\ 0 & 0.252 & 0.252 & 0.252 & 0.252 & 0.126 & 0 \\ 0 & 0 & 0.252 & 0.252 & 0.252 & 0.126 & 0.126 \\ 0.252 & 0 & 0 & 0.252 & 0.252 & 0.126 & 0.126 \\ 0.252 & 0.252 & 0 & 0 & 0.252 & 0.126 & 0.126 \end{pmatrix},$$

the difference corresponds to a cyclic shift of matrix entries. The associated spectral radii $\rho_{L=1} \approx 1.06$ and $\rho_{L=5} \approx 1.10$. Thus, the latent period determines the temporal alignment of matrix elements, which becomes relevant in the non-uniform contact scenario.

## Appendix 2. Table of spectral radii difference for combinations of infectious period and latent period for the 2 weekend days case

Recalculating the spectral radii difference for the possible combinations of infectious period $I$ and latent period $L$, we obtain Tab. A1. One population group is considered: working adults. We assume that each individual has $n_h$= 1 contact in the household daily. The contacts in the workplace $n_w$ and global setting $n_g$ are shown in Tab. 2 (uniform) and Tab.3 (non-uniform). The used infectivity values are: $p_h = 0.06$, $p_w = 0.03$ and $p_g = 0.0132$.

| L \ I | 1 | 2 | 3 | 4 | 5 | 6 | 7 | 8 | 9 | 10 |
|---|---|---|---|---|---|---|---|---|---|---|
| 0 (7) | -0.036 | 0.003 | 0.008 | 0.005 | 0.001 | 0.005 | 0 | -0.016 | -0.021 | -0.011 |
| 1 (8) | 0.009 | 0.015 | 0.015 | 0.014 | 0.017 | 0.013 | 0 | -0.004 | 0.005 | 0.012 |
| 2 (9) | 0.009 | 0.014 | 0.011 | 0.017 | 0.017 | 0.005 | 0 | 0.007 | 0.014 | 0.016 |
| 3 (10) | 0.009 | -0.010 | 0.011 | 0.014 | 0.001 | -0.005 | 0 | 0.006 | 0.008 | 0.016 |
| 4 (11) | 0.009 | 0.014 | 0.015 | 0.005 | -0.005 | -0.007 | 0 | 0.006 | 0.014 | 0.012 |
| 5 (12) | 0.009 | 0.015 | 0.008 | -0.019 | -0.022 | -0.007 | 0 | 0.007 | 0.005 | -0.011 |
| 6 (13) | 0.009 | 0.003 | -0.054 | -0.019 | -0.005 | -0.005 | 0 | -0.004 | -0.021 | -0.029 |
| $\rho_{\text{uniform}}$ | 0.216 | 0.432 | 0.648 | 0.864 | 1.080 | 1.296 | 1.512 | 1.728 | 1.944 | 2.16 |

**Table A1.** Spectral radii difference $\rho_{\text{uniform}} - \rho_{\text{non-uniform}}$ for combinations of infectious period $I$ and latent period $L$ in case of 2 weekend days. $L$ values in brackets indicate the weekly cycle. For example, the results are identical for latent periods of 0 or 7 days. In the last row, the values of $\rho_{uniform}$ are shown, which are independent of the latent period.

Spectral radii $\rho_{uniform}$ and $\rho_{non-uniform}$ values can be used for an estimate of $R_0$.

## Appendix 3. Table of spectral radii difference for combinations of infectious period and latent period for the 3 weekend days case

Recalculating the spectral radii difference for the possible combinations of infectious period $I$ and latent period $L$, we obtain Tab. A2. One population group is considered: working adults. We assume that each individual has $n_h$= 1 contact in the household daily. The contacts in the workplace $n_w$ and global setting $n_g$ are shown in Tab. 2 (uniform) and Tab. 8 (non-uniform). The used infectivity values are: $p_h = 0.06$, $p_w = 0.03$ and $p_g = 0.0132$.

| L \ I | 1 | 2 | 3 | 4 | 5 | 6 | 7 | 8 | 9 | 10 |
|---|---|---|---|---|---|---|---|---|---|---|
| 0 (7) | -0.068 | 0.002 | 0.010 | 0.019 | 0.016 | 0.012 | 0 | -0.031 | -0.044 | -0.029 |
| 1 (8) | 0.016 | 0.025 | 0.033 | 0.035 | 0.033 | 0.025 | 0 | -0.011 | 0.001 | 0.024 |
| 2 (9) | 0.016 | 0.025 | 0.032 | 0.034 | 0.033 | 0.012 | 0 | 0.007 | 0.028 | 0.041 |
| 3 (10) | 0.016 | 0.016 | 0.032 | 0.035 | 0.016 | 0 | 0 | 0.020 | 0.035 | 0.041 |
| 4 (11) | 0.016 | 0.025 | 0.033 | 0.019 | -0.012 | -0.022 | 0 | 0.020 | 0.028 | 0.024 |
| 5 (12) | 0.016 | 0.025 | 0.010 | -0.052 | -0.060 | -0.022 | 0 | 0.007 | 0.001 | -0.029 |
| 6 (13) | 0.016 | 0.002 | -0.112 | -0.052 | -0.012 | 0 | 0 | -0.011 | -0.044 | -0.063 |
| $\rho_{\text{uniform}}$ | 0.216 | 0.432 | 0.648 | 0.864 | 1.080 | 1.296 | 1.512 | 1.728 | 1.944 | 2.16 |

**Table A2.** Spectral radii difference $\rho_{\text{uniform}} - \rho_{\text{non-uniform}}$ for combinations of infectious period $I$ and latent period $L$ in case of 3 weekend days scenario as non-uniform $L$ values in brackets indicate the weekly cycle. For example, the results are identical for latent periods of 0 or 7 days. In the last row, the values of $\rho_{uniform}$ are shown, which are independent of the latent period.

Spectral radii $\rho_{uniform}$ and $\rho_{non-uniform}$ values can be used for an estimate of $R_0$.

## Appendix 4. Table of spectral radii difference for combinations of infectious period and latent period for alternating of 2 working days and 2 days off

Recalculating the spectral radii difference for the possible combinations of infectious period $I$ and latent period $L$, we obtain Tab. A3. One population group is considered: working adults. We assume that each individual has $n_h$= 1 contact in the household daily. The contacts in the workplace $n_w$ and global setting $n_g$ are shown in Tab. 2 (uniform) and Tab. 10 (non-uniform). The used infectivity values are: $p_h = 0.06$, $p_w = 0.03$ and $p_g = 0.0132$.

| L \ I | 1 | 2 | 3 | 4 | 5 | 6 | 7 | 8 | 9 | 10 |
|---|---|---|---|---|---|---|---|---|---|---|
| 0 (4) | -0.090 | 0 | 0.013 | 0 | -0.046 | -0.034 | 0.005 | 0 | -0.041 | -0.036 |
| 1 (5) | 0.019 | 0.032 | 0.029 | 0 | 0.007 | 0.037 | 0.033 | 0 | 0.004 | 0.038 |
| 2 (6) | 0.019 | 0.032 | 0.013 | 0 | 0.032 | 0.037 | 0.005 | 0 | 0.034 | 0.038 |
| 3 (7) | 0.019 | 0 | -0.050 | 0 | 0.007 | -0.034 | -0.042 | 0 | 0.004 | -0.036 |
| $\rho_{\text{uniform}}$ | 0.216 | 0.432 | 0.648 | 0.864 | 1.080 | 1.296 | 1.512 | 1.728 | 1.944 | 2.16 |

**Table A3.** Spectral radii difference $\rho_{\text{uniform}} - \rho_{\text{non-uniform}}$ for combinations of infectious period $I$ and latent period $L$ in case of 2 working days /2 days off scenario as non-uniform. $L$ values in brackets indicate the weekly cycle. For example, the results are identical for latent periods of 0 or 7 days. In the last row, the values of $\rho_{uniform}$ are shown, which are independent of the latent period.

Spectral radii $\rho_{uniform}$ and $\rho_{non-uniform}$ values can be used for an estimate of $R_0$.
